# Induced electric conductivity in organic polymers

**DOI:** 10.3762/bjnano.13.128

**Published:** 2022-12-19

**Authors:** Konstantin Yu Arutyunov, Anatoli S Gurski, Vladimir V Artemov, Alexander L Vasiliev, Azat R Yusupov, Danfis D Karamov, Alexei N Lachinov

**Affiliations:** 1 National Research University Higher School of Economics, 101000, Moscow, Russia; 2 P. L. Kapitza Institute for Physical Problems RAS,119334, Moscow, Russia; 3 Federal research center “Crystallography and photonics” RAS, 119333, Moscow, Russia; 4 National research center “Kurchatov institute”, 123182, Moscow, Russia; 5 Moscow Institute of Physics and Technology (State University), MIPT, 141701 Moscow Region, Russia; 6 Bashkir State Pedagogical University n.a. M. Akmulla, 450008, Ufa, Russia; 7 Institute of Molecule and Crystal Physics UFRC RAS, 450054, Ufa, Russia

**Keywords:** conducting polymer, superconductivity, thin films

## Abstract

Poly(diphenylene phthalide) (PDP) belongs to the class of carbocyclic organic electroactive polymers, which exhibits electric conductive properties when an external electric field and/or mechanical stress is applied. In this work, the transport properties of thin-film layered lead–PDP–lead structures were experimentally studied in a wide temperature range. At sufficiently high temperatures, the current voltage characteristics are satisfactorily described in terms of the injection model of currents limited by the space charge. At temperatures below ≈8 K, a number of samples exhibit features that can be explained by the effect of induced superconductivity in a thin film of conducting polymer enclosed between two massive superconductors (lead).

## Introduction

Most polymers can be classified as organic dielectrics. However, there exists a specific class of polymers, typically characterized by the existence of conjugated π-bonds, which enable delocalization of electrons leading to electric conductivity in the ground state of the system. Relatively recently it was found that finite electric current can pass also through non-conjugated polymers. In the ground state they are wide-band dielectrics, but can exhibit high electric conductivity under the influence of such external parameters as mechanical stress and/or electric field [1]. The effect is interpreted as stimulation of metallic state [2].

**Figure 1:**
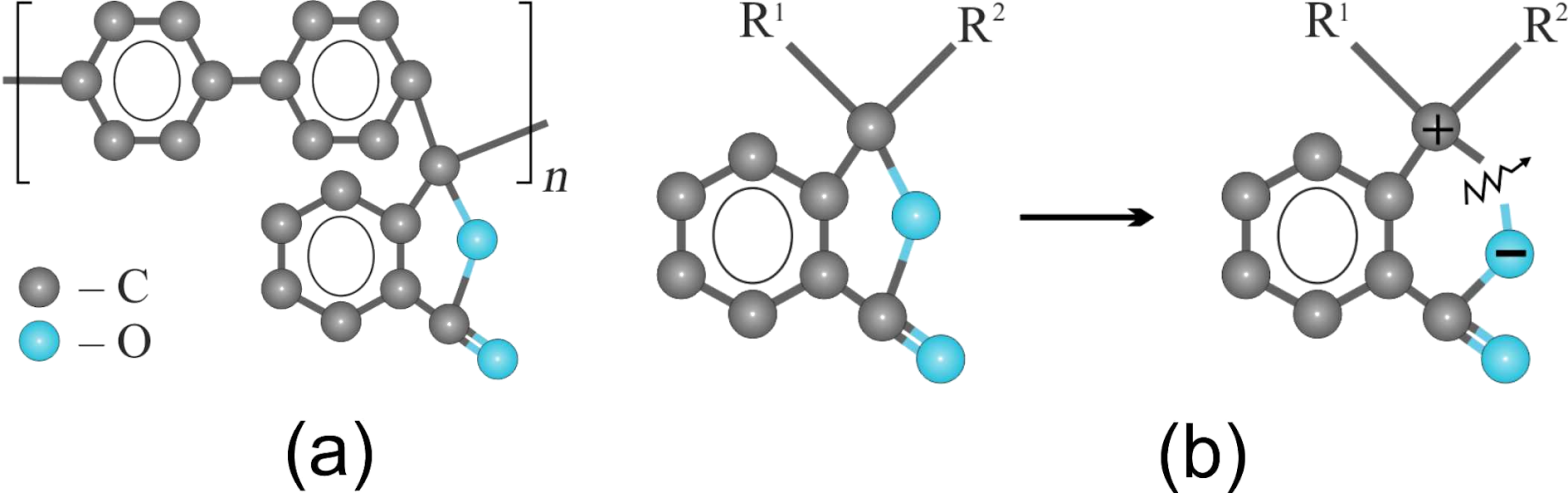
(a) Structural unit of poly(diphenylene phthalide) (PDP) molecule. (b) Schematics of C–O bond break leading to C^+^ and O^−^ ionization of the corresponding atoms, which enables effective charge transfer along the polymer chain [2].

Poly(diphenylene phthalide) (PDP) was chosen as the object of study being a representative electro-active polymer demonstrating induced electric conductivity. PDP is a carbocyclic polymer with high chemical and thermal stability, transparency in the visible spectrum, and high mechanical strength [3–4]. PDP has an amorphous structure with a degree of crystallinity not exceeding 15%. It is characterized by unusually high chemical resistance. The conditions for the selective production of PDP with a molecular weight of more than (50–60) × 10^3^ without the formation of a gel fraction have been found. A model image of the structural unit of the PDP polymer is shown in Figure 1a. Polymers of this class are soluble in organic solvents and have exceptional film-forming properties [5–6]. According to these results, continuous homogeneous films can be obtained by centrifugation from a solution in cyclohexanone on a metal surface with a thickness from several nanometers up to micrometers. High homogeneity and defect-free surfaces on nanoscopic scales have been repeatedly confirmed by various methods, including scanning tunneling and atomic force microscopy. In some cases, it was possible to observe regions with surface macromolecular (quasicrystalline) ordering [7].

The remarkable property of PDP is that, depending on the length of certain atomic bonds, its molecule can exist in several spatial configurations. Under normal conditions, PDP is a wide-gap dielectric material and is characterized by the following parameters: band gap ≈4.3 eV, electronic work function ≈4.2 eV, electron affinity ≈2 eV, first ionization potential ≈6.2 eV. Experimental evaluations of the electronic parameters of PDP have been made earlier by various methods [8–9]. Quantum chemical studies of PDP [10] have shown that its molecular structure is unstable with regard to interaction with an excess (thermal) electron and can result in a transition to a metastable state. However, in that state, e.g. induced by external electric field, the system is characterized by non-zero density of electronic states within the energy gap. The depth of such states increases if the system accepts an extra electron (Figure 1b), thus indirectly enabling electric conductivity along the polymer chain [11]. Later the validity of this model was supported experimentally and further elaborated [12–14].

The purpose of this work was to study this interesting phenomenon in a wide temperature range: from 4.2 K to room temperature.

## Experimental

**Figure 2:**
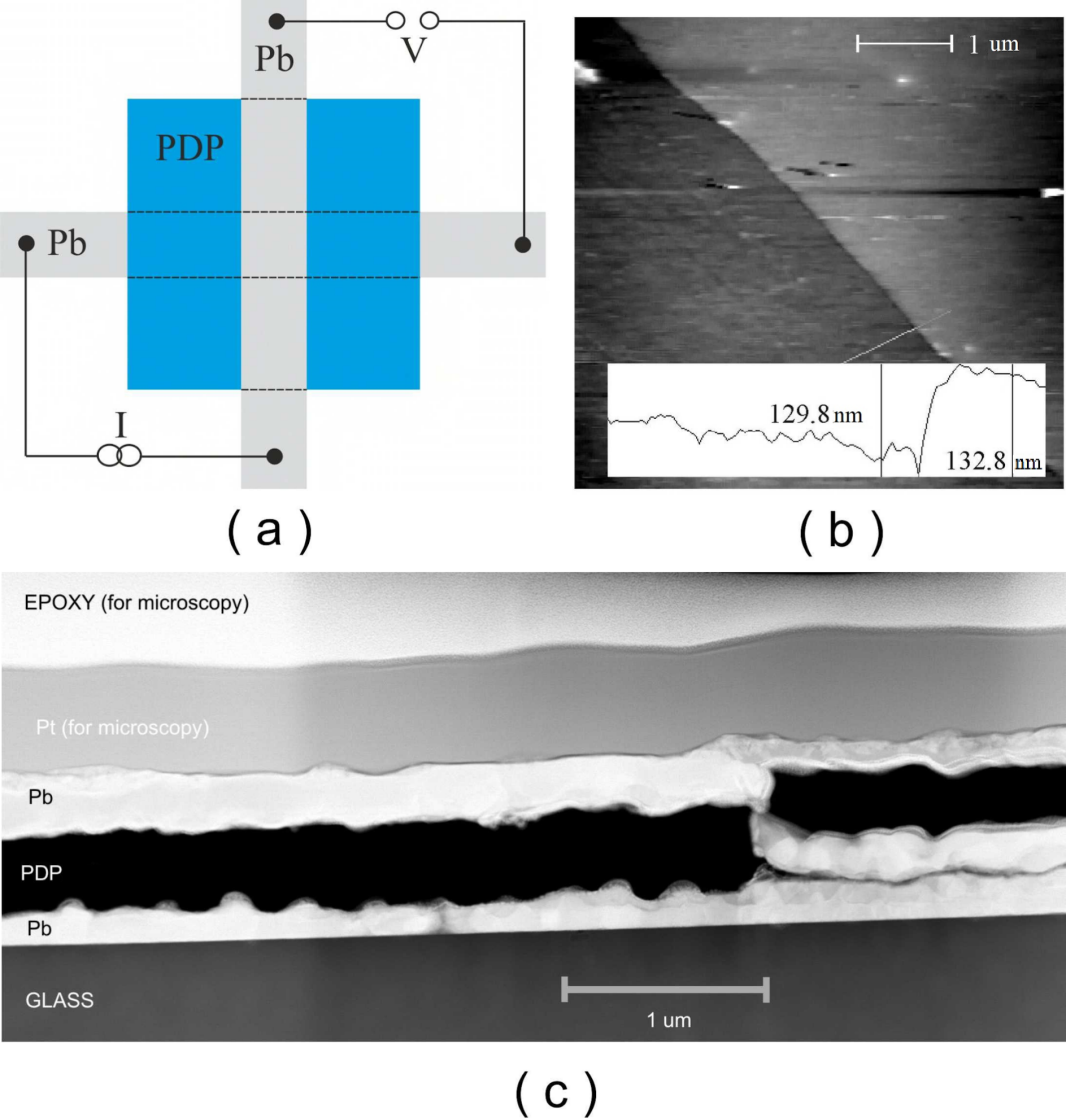
(a) Schematics of a Pb (grey)–PDP (blue)–Pb (grey) three layer heterostructure on insulating substrate (not indicated for simplicity). The layout enables electron transport measurements of each lead strip and the whole sandwich itself. (b) Atomic force microscope scan of a PDP film 0.1 wt % on Si substrate. The plot at the bottom illustrates the roughness of the surface along the indicated line. (c) Side view of a Pb–PDP–Pb structure on glass with solitary defect (lead shortcut) obtained by transmission electron microscopy.

Layered Pb–PDP–Pb heterostructures were fabricated in a glove box in nitrogen atmosphere with minimum moisture and oxygen content without exposure to room atmosphere between cycles of formation of different layers. Glass or oxidized silicon were used as substrates. The substrates were preliminarily cleaned in ethanol and distilled water in an ultrasonic bath. The surfaces were hydrophilized by treating the substrates with cyclohexanone immediately before applying the polymer solution. The electrodes consisted of two mutually perpendicular lead strips with a width of ≈1 mm, between which the PDP polymer film was ‘sandwiched’ (Figure 2a). Lead was chosen as fairly low-melting metal exhibiting superconducting properties. Formation of thin-film lead electrodes with thicknesses from 50 nm to 200 nm was carried out by thermal evaporation in vacuum. The critical temperature of bulk lead is *Т*_с_(Pb_3D_) = 7.2 K. However, in the form of a thin film, the critical temperature of a superconductor can differ significantly from the tabulated value [15–16]. In our samples, the critical temperature of lead electrodes varied from 7.8 K < *T*_c_(Pb_film_) < 8.2 K. Submicron PDP films were prepared by centrifuging the polymer from a solution in cyclohexanone on a solid substrate. When preparing the solution, the polymer was first soaked in a small amount of solvent until complete dissolution, then the solvent was added in the required amount, and the solution was kept for another day in the dark. For fabrication of films of various thicknesses, solutions of polymers in cyclohexanone with concentrations of 0.1–15 wt % were used. The polymer solution of specified concentration was applied onto the dielectric substrate fixed on a centrifuge holder. The rotation speed was typically 2000 rpm. The resulting polymer film was dried in air for about 45–60 min at room temperature. Then the final drying was carried out to remove solvent residues at a temperature of 150–200 °C for 45 min. Depending on the concentration of the solutions, it was possible to obtain films of various thicknesses from several nm to several μm. The formation of films from dilute solutions occurs according to the mechanism of linear or loop adsorption, when macromolecules at the interface are completely or partially elongated. Also, film formation at such low concentrations strongly depends on the energy interaction of macromolecules with the substrate surface, which explains the weak dependence of the film thickness on the solution concentration. With an increase in concentration, associates of macromolecules are formed in the solution, and the influence of adhesion processes decreases, but the cohesive forces increase. In the entire thickness range from 3 nm to 1 µm, the films are solid, without significant defects and/or pin holes. The polymer films were studied by atomic force microscopy (AFM) using an earlier described methodology [5]. The study of the film morphology showed that they are homogeneous, and within the entire thickness range from 3 nm to 1 µm the films are solid, without significant defects and/or pin holes. The observation confirms the good film-forming properties of the PDP polymer solutions. For example, Figure 2b shows the topography of the polymer film 0.1 wt % on Si substrate. The plot at the bottom demonstrates the variation of the structure along the line, depicted at the upper panel. The distance between the measuring lines is of the order of the polymer film thickness, which is just 3 nm.

After electric measurements, a number of heterostructures was sent for analysis by high resolution transmission electron microscopy and/or scanning electron microscopy. None of the studied samples showed a systematic ‘sticking’ of lead electrodes through the thickness of the polymer or formation of multiple metallic dendrites. However, a number of microphotographs showed some defects in the form of shortcuts (Figure 2c). The origin of these artifacts is not entirely clear: they could be initially present in heterostructures, or they could have appeared during the preparation of a sample for electron microscopy.

## Results and Discussion

The experiment was carried out in a four-contact configuration at direct or alternating currents. Both *R*(*T*) and *V*(*I*) dependences of the Pb–PDP–Pb sandwich could be measured, as shown in Figure 2a, and the transport characteristics of each lead electrode separately. To measure differential characteristics d*I*/d*V*(*V*), modulation technique and phase-sensitive lock-in detection were used. To suppress the negative effect of stray electromagnetic pickups, a multistage RLC filter system was used [17]. While *R*(*T*) measurements at cryogenic temperatures, the current value from 0.1 to 100 μA was chosen so that its increase by an order of magnitude would not lead to a noticeable shift in the temperature of the superconducting phase transition. All experiments were carried out in a ^4^He direct pumped cryostat. The semiconducting thermosensors were calibrated by the ^4^He vapor pressure and by the reference points of superconducting transitions in pure bulk superconductors (Al, In, Sn, Pb). The resulting absolute error in determining the temperature was ≈10 mK, while the relative error was less than 1 mK. Repeated measurements of *T*_c_ of the same sample coincided with an accuracy of several mK. An analysis was made of the degradation of samples over time. The difference between two measurements of the same sample was 3 months, while the shift in the beginning of the phase transition was minimal δ*Т*_с_ ≈ 0.01 K.

It has been established that in the measured Pb–PDP–Pb structure, the shape of the current–voltage characteristics strongly depends on temperature. At 300 K the *I*–*V* dependencies have a nonlinear character *j* ≈ *kU^n^*, typical for organic dielectrics. At temperatures ≈77 K and below, the dependence *j* = *f*(*U*) is also nonlinear, but is significantly different. In particular, as the voltage increases, there is the tendency for the current flowing through the heterostructure to saturate. Previously [2], it was found that at high temperatures, the mechanism of overcoming the barrier at the metal/polymer contact is satisfactorily described in terms of the injection current model limited by the space charge. At low temperatures, the tunneling mechanism is the predominant mechanism.

**Figure 3:**
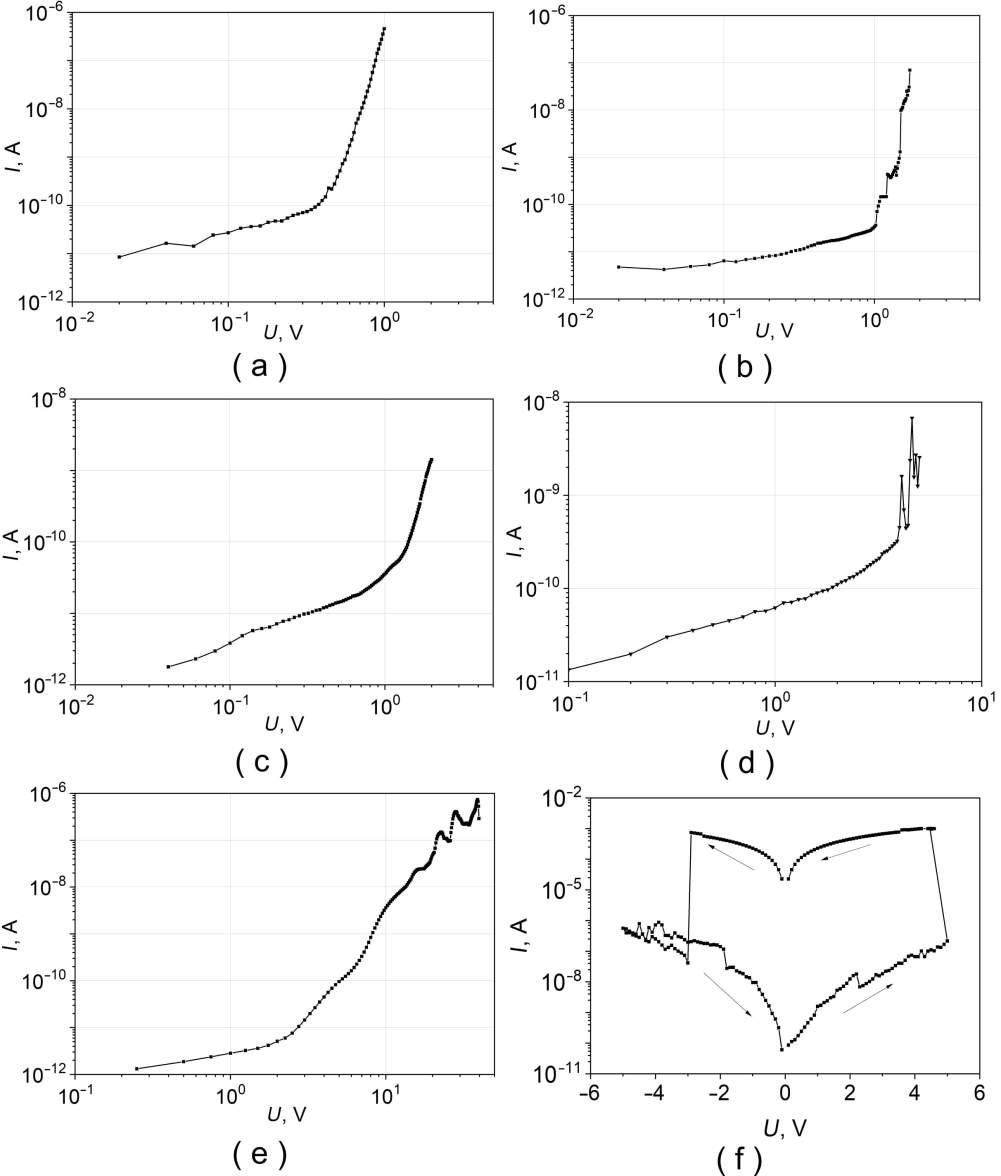
Current–voltage characteristics, measured at room temperature, of several Pb–PDP–Pb sandwiches with different thicknesses of the polymer film: (a) 10 nm, (b) 25 nm, (c) 45 nm, (d) 150 nm, (e) 360 nm; (f) is the same structure as in (c) and with 2 kOhm load resistance in the electric circuit; (f) demonstrates the switching phenomenon of the thin PDP film from insulating to metal state. Thickness of Pb electrodes is about 200 nm.

Figure 3 shows the current–voltage characteristics of Pb–PDP–Pb structures with different PDP film thicknesses. With increase of the polymer film thickness, the current decreases. Therefore, they were measured over a wide voltage range. The shape of the *I*–*V* characteristics depends on the energy spectrum. Several regimes could be distinguished on the *I*–*V* plots: (a) ohmic mode at low voltages: thermally generated charge carriers prevail; (b) mode with a predominance of traps: trapped charge carriers in small traps limit the current; (c) the limit of trap filling: the quasi-Fermi level reaches the energy of the trap *E*_t_, the traps are filled and there is a steep increase in current. It should be noted that in a circuit with finite load resistance the effect of electronic switching to a highly conductive state can be observed (Figure 3f). Such a bi-stable switching is often used in practice to create non-volatile memory elements.

Presented in Figure 3 *I*–*V* characteristics do not contradict the presented above model considerations. In this regard, to analyze the *I*–*V* at 300 K, one can apply the formalism of the theory of injection currents, which makes it possible to estimate the concentration of intrinsic charge carriers (*n*_0_) and their minimum mobility (μ) [18]:


[1] $$n_{0}=\frac{\varepsilon \varepsilon_{0}U_{n}}{eL^{2}}$$

[2] $$\mu =\frac{jL^{3}}{\varepsilon \varepsilon_{0}U_{n}^{2}}$$

where, *j* is the current density, *L* is the distance between the electrodes, *U_n_* is the voltage corresponding to the transition point from linear to parabolic dependency of the *I*–*V*’s, *n*_0_ is the equilibrium concentration of charge carriers, ε and ε_0_ dielectric constants of the polymer and vacuum, respectively, and μ is the maximum mobility of charge carriers.

According to this model, the ohmic behavior of the *I*–*V* characteristics at low voltages is due to intrinsic charge carriers. If, however, the concentration of injected carriers begins to exceed the concentration of intrinsic carriers, then the transition to a sublinear regime is observed. The corresponding estimations give the following values *n*_0_ = 10^21^−10^23^ m^−3^, μ = 10^−15^ to 10^−17^ m^2^/Vs.

The analysis of the *I*–*V* characteristics within the framework of Schottky barrier formation makes it possible to estimate the height of potential barriers at the metal/polymer interfaces utilizing the Richardson expression [18]:


[3] $$\varphi =\frac{kT}{e}\ln\left(\frac{AA*T^{2}}{I_{\text{s}}}\right)$$

where *T* is the temperature, *k* is the Boltzmann constant, *e* is the electron charge, *A* is the contact area, A* is the Richardson constant*, I*_s_ the saturation current. To proceed, it is important to determine the saturation current using, e.g., the semi-logarithmic dependence of the current on applied voltage. This is the so-called current at zero voltage. In addition, it is necessary to take into account the non-ideality coefficient of the barrier. We chose the value of the latter from previous measurements. As the result, the value of the potential barrier calculated using Equation 3 is equal to 0.7. As expected, the transport characteristics of thin-film lead electrodes demonstrated the metallic behavior: in normal state, the resistance of the films decreased linearly with decreasing temperature, and the *I*–*V* characteristics exhibit ohmic behavior. At temperatures of the order of *T*_c_(Pb_film_) ≈ 8 K, a sharp superconducting transition was observed.

**Figure 4:**
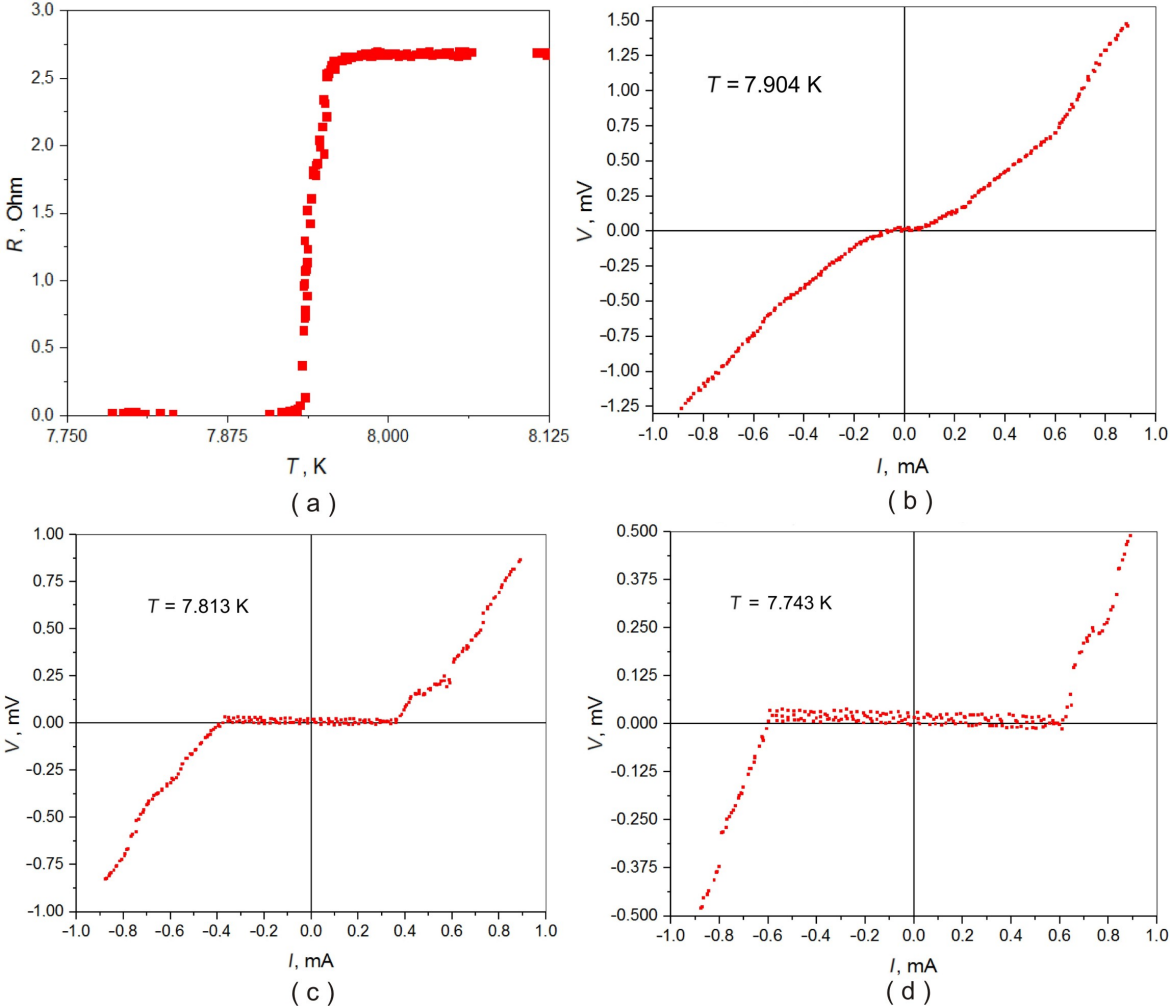
Sandwich structure Pb–PDP–Pb–0.4-GLASS, thickness of polymer is about 350 nm, both Pb electrodes are ≈200 nm thick. (a) Resistance vs temperature *R*(*T*) dependence; (b), (c), and (d) voltage vs current *V*(*I*) characteristics measured at various temperatures.

Perhaps the most interesting are the results of transport measurements of Pb–PDP–Pb sandwiches at temperatures below the critical temperature of the superconducting transition of lead electrodes. In a number of samples, with a polymer film thickness of less than 350 nm, features are observed that unambiguously indicate the presence of a superconducting state: a sharp drop in resistance to instrumental zero ≈5 nV (Figure 4a) and presence of supercurrent in the *I*–*V*’s (Figure 4b,c and d). A trivial explanation may be the occurrence of shortcuts between the lead plates: either macroscopic ‘pinholes’ or formation of multiple thin dendrites. The second possibility seems unlikely: neither the previous studies, nor the selective microscopic analysis of several samples by scanning and transmission electron microscopy revealed signs of the presence of dendrites. While the side view microphotographs made by high-resolution transmission electron microscopy occasionally reveal some macroscopic features such as lead electrode shortcuts through the polymer film (Figure 2c). These defects are episodic, and their character does not resemble the ‘melting through’ of the PDP film during the thermal deposition of lead. Moreover, the PDP decomposition starts at 440 °С, which is significantly higher than the melting point of lead, 327 °C. Moreover, it cannot be ruled out that such defects as ‘collapse’ of lead electrodes (Figure 2c) are not intrinsic, and could have appeared only during the process of sample preparation for transmission microscopy. The sufficiently high critical current (Figure 4b–d) also indicates the large area of the conductive channel, which does not correspond to the scenario of the occurrence of single point short circuits. The *I*–*V* dependencies of the Pb–PDP–Pb sandwiches above the superconducting temperature of the lead electrodes are essentially non-ohmic (Figure 3), which supports the claim about the absence of trivial metal-to-metal shortcuts.

The second alternative explanation of the dependencies in Figure 4 may be the Josephson effect: the flow of supercurrent between two superconductors separated by a dielectric barrier. However, the correlation of the order parameters of two spatially separated superconductors is a subtle quantum mechanical effect, which in all practical cases is observed at dielectric thicknesses of the order of several nm, while in our case superconductivity in Pb–PDP–Pb sandwiches manifests itself at polymer thicknesses up to 350 nm. Hence, it can be assumed that the dependencies in Figure 4 can be explained by the effect of induced superconductivity in a thin film of conducting polymer enclosed between two massive superconductors (lead). The substantiation of this assertion requires further verification. In the near future, experiments are planned on planar heterostructures, where superconductivity, if observed, cannot be explained by trivial metallic shortcuts.

## Conclusion

*R*(*T*), *V*(*I*) and d*V*/d*I*(*V*) dependences of thin-film layered structures lead–PDP–lead were experimentally studied in a wide temperature range. At sufficiently high temperatures, the *I*–*V* dependences are satisfactorily described in terms of the currents injection model limited by the space charge. At temperatures below ≈8 K, a number of samples exhibit features that can be explained by the effect of induced superconductivity in a thin film of a conducting polymer enclosed between two massive superconductors (lead).
